# The soluble epoxide hydrolase inhibitor TPPU improves comorbidity of chronic pain and depression via the AHR and TSPO signaling

**DOI:** 10.1186/s12967-023-03917-x

**Published:** 2023-02-02

**Authors:** Ailin Luo, Zifeng Wu, Shan Li, Cindy B. McReynolds, Di Wang, Hanyu Liu, Chaoli Huang, Teng He, Xinying Zhang, Yuanyuan Wang, Cunming Liu, Bruce D. Hammock, Kenji Hashimoto, Chun Yang

**Affiliations:** 1 Department of Anesthesiology, Tongji Hospital, Tongji Medical College, Huazhong University of Science and Technology, Wuhan, 430030 China; 2 Department of Anesthesiology and Perioperative Medicine, The First Affiliated Hospital of Nanjing Medical University, Nanjing, 210029 China; 3 State Key Laboratory of Pharmaceutical Biotechnology, Model Animal Research Center, Nanjing University, Nanjing, 210061 China; 4 Department of Entomology and Nematology and UC Davis Comprehensive Cancer Center, University of California, Davis, CA 95616 USA; 5 Division of Clinical Neuroscience, Chiba University Center for Forensic Mental Health, Chiba, 260-8670 Japan

**Keywords:** Chronic pain, Depression, Soluble epoxide hydrolase, TPPU, Translocator protein, Aryl hydrocarbon receptor

## Abstract

**Background:**

Patients suffering from chronic pain often also exhibit depression symptoms. Soluble epoxide hydrolase (sEH) inhibitors can decrease blood levels of inflammatory cytokines. However, whether inhibiting sEH signaling is beneficial for the comorbidity of pain and depression is unknown.

**Methods:**

According to a sucrose preference test (SPT), spared nerve injury (SNI) mice were classified into pain with or without an anhedonia phenotype. Then, sEH protein expression and inflammatory cytokines were assessed in selected tissues. Furthermore, we used sEH inhibitor TPPU to determine the role of sEH in chronic pain and depression. Importantly, agonists and antagonists of aryl hydrocarbon receptor (AHR) and translocator protein (TSPO) were used to explore the pathogenesis of sEH signaling.

**Results:**

In anhedonia-susceptible mice, the tissue levels of sEH were significantly increased in the medial prefrontal cortex (mPFC), hippocampus, spinal cord, liver, kidney, and gut. Importantly, serum CYP1A1 and inflammatory cytokines, such as interleukin 1β (IL-1β) and the tumor necrosis factor α (TNF-α), were increased simultaneously. TPPU improved the scores of mechanical withdrawal threshold (MWT) and SPT, and decreased the levels of serum CYP1A1 and inflammatory cytokines. AHR antagonist relieved the anhedonia behaviors but not the algesia behaviors in anhedonia-susceptible mice, whereas an AHR agonist abolished the antidepressant-like effect of TPPU. In addition, a TSPO agonist exerted a similar therapeutic effect to that of TPPU, whereas pretreatment with a TSPO antagonist abolished the antidepressant-like and analgesic effects of TPPU.

**Conclusions:**

sEH underlies the mechanisms of the comorbidity of chronic pain and depression and that TPPU exerts a beneficial effect on anhedonia behaviors in a pain model via AHR and TSPO signaling.

**Supplementary Information:**

The online version contains supplementary material available at 10.1186/s12967-023-03917-x.

## Introduction

Pain is an unpleasant feeling or emotional experience accompanied by actual or potential damage to tissues, organs, and nerves [1]. Chronic pain frequently lasts more than three months, is difficult to cure, and is an intractable clinical issue. Depression is a common mental disease that mainly manifests as low mood, anhedonia, and even cognitive impairment [2]. Interestingly, depression is often found accompanying with chronic pain, and depressive symptoms aggravate pain severity and duration. However, the pathophysiology of the comorbidity of chronic pain and depression remains elusive [3, 4]. In this regard, the full elucidation of the mechanisms underlying the comorbidity of chronic pain and depression and the adoption of effective therapeutic strategies are important.

Inflammation plays an important role in developing both pain and depression [5, 6]. Inflammatory cytokines and chemokines act on nociceptors and transmit the deleterious stimulus to the central nervous system, resulting in abnormal or overactivated neuroinflammation [7, 8]. In addition, inflammatory cytokines also appear in the cortices and hippocampi of brain tissues in patients with major depressive disorder [9]. The hyperactivity and hyperexcitability of neurons characterize central sensitization in the brain and spinal cord, originating from persistent nociceptive input during chronic pain [8]. Therefore, inflammation is likely to play a critical role in the pathogenesis of chronic pain and depression. Resolving the overactivated inflammatory system might benefit the recovery of the comorbidity of pain and depression.

The metabolism of arachidonic acid (ARA) via cytochrome P450s (CYP450s) produces epoxy fatty acids (EpFAs), which provide protective effects in inflammatory disorders [10]. Eicosanoids are metabolites of ARA and other long chain fatty acids. The long fatty acids can be epoxidized by CYP450 to produce four regioisomeric epoxyeicosatrienoic acids (EETs) (i.e., 5,6-EET, 8,9-EET, 11,12-EET, and 14,15-EET). Moreover, omega-3 polyunsaturated fatty acids (PUFAs), such as eicosapentaenoic acid (EPA) and docosahexaenoic acid (DHA), can also be catalyzed into epoxyeicosatetraenoic acids (EEQs) and epoxydocosapentaenoic acids (EDPs),collectively referred to as epoxy-fatty acids (EpFAs) [11]. EETs and other EpFAs can reduce the release of the vascular cell adhesion molecule 1 (VCAM-1), intercellular adhesion molecule 1 (ICAM-1), and endothelial cell-selective adhesion molecule (E-selectin). They can also inhibit the nuclear factor κB (NF-κB) signaling pathway, exerting anti-inflammatory and neuroprotective effects [12–14]. Soluble epoxide hydrolase (sEH, encoded by the *Ephx2* gene) catalyzes the hydrolysis of EETs to dihydroxyeicosatrienoic acids (DHETs) with lower biologically activity [15] and far greater polarity. In the human brain, sEH occurs in neurons, oligodendrocytes, astrocytes, and ependymal cells [16]. Numerous studies have shown that sEH inhibitors (sEHIs) can treat neuropathic pain and inflammatory pain [17, 18]. Importantly, sEH levels in the parietal cortex were higher in patients with major depressive disorders compared with control subjects [19]. sEHI improved the behavioral performance of mice with lipopolysaccharide (LPS)-induced depression-like behavior and induced the release of the regulatory neurotrophic factor [20, 21]. Therefore, therapeutic strategies aimed at inhibiting sEH might have the pharmacological benefit of curing chronic pain–depression comorbidity.

sEH helps transform the anti-inflammatory products of CYP epoxidation into inactive diols [22]. CYPs comprise various subfamilies, such as CYP1A, CYP1B, etc. [23]. The aryl hydrocarbon receptor (AHR) is a transcription factor expressed in the cytosol which translocates to the nucleus after binding to its ligands. After dimerization with the AHR nuclear translocator (ARNT), AHR/ARNT dimers bind to the enhancer region of the *CYP1A1* and other genes [24, 25]. In the metabolism of PUFAs, multiple CYPs produce EETs by epoxidation, as well as hydroxylation of ARA and other PUFAs, to pro-inflammatory hydroxyeicosatetraenoic acids (HETEs) [26, 27]. In association with immune/inflammatory diseases, an increased AHR level appears in patients with major depression and multiple sclerosis [28]. Excessive activation of AHR impaired the differentiation and proliferation of hippocampal neural progenitor cells, thus affecting neurogenesis and cognitive function [29]. Collectively, these findings indicate that the sEH–AHR signaling pathway may be involved in pro-inflammatory effects by regulating the metabolism of PUFAs.

In contrast to sEH and AHR, the translocator protein (TSPO) plays an anti-inflammatory role by transporting cholesterol [30]. TSPO is located in the outer mitochondrial membrane and transports cholesterol to the inner membrane, leading to the synthesis of neurosteroids in the CNS [31]. The activation of TSPO ameliorates chronic inflammatory and neuropathic pain [32, 33]. Increased cholesterol levels in the brain are frequently associated with neurodegenerative diseases and neuroinflammation [34]. In turn, the normalization of cholesterol metabolism in spinal microglia reduced the release of inflammatory cytokines and relieved chronic neuropathic pain [35]. Moreover, the TSPO ligand antagonizes the neurotoxic effects mediated by *N*-methyl-d-aspartate receptors, inducing antidepressant and antianxiety effects [36]. Interestingly, EETs exerted a cholesterol-lowering effect, and both *Ephx2* knockout and sEHI reduced plasma cholesterol levels [37, 38].

The purpose of the present study was to elucidate the role of sEH in the chronicpain–depression comorbidity induced by SNI surgery. We planned to investigate the effects of the sEH inhibitor TPPU on post-operative symptoms of hyperalgesia and anhedonia and detect changes in TSPO and AHR levels in selected tissues. This study’s findings suggest that the alleviation of the pain–depression comorbidity by sEH inhibitors occurs via distinct signaling pathways involving TSPO or AHR. This will provide important tools and approaches for studying mechanisms underlying the similarities and differences of the comorbidity of pain and depression.

## Materials and methods

### Animals

Two month-old male C57BL/6 mice (body weight 20–25 g) were purchased from the Animal Center of Tongji Hospital in this study. Animals were acclimated to the environmental conditions for 7 days before the experiment. Animals were housed in controlled temperature (22 ± 2 °C.) and 12 h light/dark cycles (lights on 8:00) with food and water ad libitum. The study was conducted in strict accordance with the recommendations in the Guide for the Care and Use of Laboratory Animals and under protocols approved by the Experimental Animal Committee of Tongji Hospital, Tongji Medical College, Huazhong University of Science and Technology (Wuhan, China).

### Spared nerve injury

The SNI surgery was performed as previous described [39]. Briefly, the 3 peripheral branches (sural, common peroneal, and tibial nerves) of left sciatic nerve were exposed under general anesthesia of 1.5–2.5% isoflurane. The tibial and common peroneal nerves were ligated with 6-0 silk suture and cut off the distal to the ligation, while nerves were exposed but not transected for sham surgery.

### Behavioral tests

All behavioral procedures were conducted in the dark phase (active for mice) of the daily light/dark cycle. To reduce the influence of artificial factors on results, the individual who carried out the tests and data analysis was blind to the experimental assignments, and all animals were tested under consistent conditions and using the same apparatus.

### MWT

Before MWT, mice were placed in plexiglass chambers with a wire net floor for 30 min in consecutive 6 days to acclimate to the test conditions. Mechanical allodynia was measured as previously described [40, 41]. The Von Frey monoflaments were applied to the lateral 1/3 of left paws using the up-and-down method. The paws quick withdrawal or flinching was considered as a positive response. All filament stimuli were applied 4 times with a period of 30 s interval.

### SPT

Mice were feed separately and exposed to two identical bottles (tap water and 1% sucrose solution) for 48 h, and the bottles were exchanged every 24 h to avoid place preference. Mice were followed by 24 h of water and food deprivation and 24 h exposure to two identical bottles. The bottles containing water and sucrose were weighed before and at the end of this period and the sucrose preference was determined.

### Experiment design

#### Study 1: SNI on mechanical allodynia, anhedonia-like symptoms and sEH protein expression

To assess the effects of chronic pain on anhedonia and sEH protein expression, 26 mice were randomly divided into two groups (Sham, n = 8; SNI, n = 18), baseline threshold of mechanical allodynia was undertaken on 1 day before SNI and re-measured on day 7, 14, 21 after surgery. Sucrose preference test (SPT) was performed on day 5, 12 and 19 after SNI to assess the anhedonia-like symptoms. On day 22 after surgery, clustering analysis according to SPT scores was performed to divide SNI mice into SNI with anhedonia susceptible group (Anhedonia susceptible, n = 8) and SNI without anhedonia (Anhedonia resilient, n = 10). Samples were collected on day 23 postoperatively after anesthesia of isoflurane.

#### Study 2: TPPU alleviated mechanical allodynia and anhedonia-like symptoms associated with TSPO signaling and AHR signaling

To determine the effects of TPPU on mechanical allodynia and anhedonia, 56 mice were randomly assigned two groups (sham, n = 16; SNI, n = 40). On day 15, clustering analysis according to SPT scores was performed to divide SNI mice into SNI with anhedonia susceptible group (Anhedonia susceptible; n = 16) and SNI without anhedonia (Anhedonia resilient; n = 24). Vehicle (30% PEG 400) or TPPU (3 mg/kg, dissolved in PEG 400), a sEH inhibitor, was administered orally to one of four groups (n = 8 per group): sham group treated daily with vehicle or TPPU, anhedonia susceptible mice with vehicle or TPPU. MWT was performed 30 min after consecutive 7 days treatment. SPT was undertaken to evaluate the therapeutic effects of TPPU on anhedonia-like symptoms 21 days after surgery. Samples were collected on day 23 postoperatively after anesthesia of isoflurane.

#### Study 3: AHR agonist offset the antidepressant but not analgesia effects of TPPU

To determine the role of AHR in the therapeutic effects of TPPU on chronic pain and anhedonia, 66 mice were divided into two groups (Sham, n = 6; SNI, n = 60). Similarly, anhedonia susceptible mice selected from SNI group by clustering with SPT scores, and then were randomly assigned to 4 groups: anhedonia susceptible without any compounds (Sus, n = 6), anhedonia susceptible daily treated with CH-223191(10 mg/kg, dissolved in corn oil) from day 15 for consecutive 7 days (Sus + CH-223191, n = 6), anhedonia susceptible treated with FICZ (100 ug/kg, dissolved in corn oil) on day 15 (Sus + FICZ, n = 6), and anhedonia susceptible simultaneously treated with FICZ and TPPU on day 15, thereafter treated with TPPU solely for consecutive 6 days (Sus + FICZ + TPPU, n = 6). Behavioral tests were performed as the same as study 3. Samples were collected on day 23 postoperatively after anesthesia of isoflurane.

#### Study 4: TSPO antagonists offset the antidepressant and analgesia effects of TPPU

To determine the role of TSPO in the therapeutic effects of TPPU on chronic pain and anhedonia, 110 mice were divided into two groups (Sham, n = 8; SNI, n = 102). Similarly, anhedonia susceptible mice selected from SNI group by clustering with SPT scores, and then were randomly assigned into 4 groups: anhedonia susceptible without any compounds (Sus, n = 8), anhedonia susceptible daily treated with TPPU (3 mg/kg, dissolved in PEG 400) from day 15 for consecutive 7 days (Sus + TPPU, n = 8), anhedonia susceptible treated with Finasteride (10 mg/kg, dissolved in corn oil), TPPU (3 mg/kg) from day 15 for consecutive 7 days (Sus + Fina + TPPU, n = 8), and anhedonia susceptible simultaneously treated with PK-11195 (3 mg/kg, dissolved in 2% DMSO and 0.8% Tween) and TPPU (3 mg/kg) from day 15 to day 21 (Sus + PK + TPPU, n = 8), anheonia susceptible with AC5216 (1 mg/kg, suspended in 0.5% tragacanth gum aqueous solution) from day 15 to day 21 (Sus + AC, n = 8). Behavioral tests were performed as the same as study 2 or study 3. Samples were collected on day 23 postoperatively after anesthesia of isoflurane.

### Drug administration

The sEH inhibitor TPPU was synthesized at Professor Bruce Hammock’s lab (University of California, Davis). TPPU (3 mg/kg) was dissolved in 20% (vol/vol) polyethylene 400 (PEG 400, Cat#P8530, Solarbio, Beijing) [21]. The AHR inhibitor CH-223191 (10 mg/kg, HY-12684, MedChemExpress, USA) and AHR agonist FICZ (100 ug/kg, HY-12451, MedChemExpress, USA) were dissolved in corn oil and intragastrically administered as previously described [42, 43]. TSPO antagonist PK-11195 (3 mg/kg, ab120378, Abcam, UK) was dissolved in 2% DMSO and 0.8% Tween and then diluted by saline. Finasteride (10 mg/kg, HY-13635, MedChemExpress, USA), a 5α-reductase inhibitor, was dissolved in corn oil. The above two regents were previously reported to block the effects of TSPO overexpression [44]. AC-5216 (1 mg/kg, HY-15527, MedChemExpress, USA) were suspended in 0.5% tragacanth gum aqueous (CAT#G9390, Solarbio, Beijing) solution for oral administration, which was reported to induce antianxiety and antidepressant effects [45].

### Western blot analysis

As described previously, tissues were grated and homogenized with RIPA buffer (Boster, Wuhan, China) at 4 °C for 30 min, then centrifuged and supernatants were collected. The protein concentrations in supernatants were determined by BCA protein assay kit (Boster, Wuhan, China). The protein samples were separated by 10% sodium dodecyl sulfate–polyacrylamide gel (SDS-PAGE) electrophoresis, and then were transferred to poly vinylidene fluoride (PVDF) membranes (Millipore, Bedford, MA, USA). Bands were blocked with 5% BSA dissolved in TBST (0.1%Tween 20 in Tris-buffered saline) for 1 h at room temperature. Relative primary antibodies were incubated at 4 °C overnight: rabbit EPHX-2 (1:5000, ab155280, Abcam), rabbit AHR (1:500, AF6278, Affinity), rabbit TSPO (1:1000, DF8227, Affinity), and rabbit GAPDH (1:5000, AF7021, Affinity,). After warming and washing by TBST, second antibody was incubated on bands for 2 h at room temperature: goat anti-rabbit IgG horseradish peroxidase (1:5000, Promotor, Wuhan, China). Finally, these protein bands were visualized by enhanced chemiluminescence substrate solutions (Promotor, Wuhan, China) with the ChemiDoc XRS chemiluminescence imaging system (Bio-Rad, Hercules, CA, USA).

### ELISA

The IL-6, IL-1β, TNF- α, CYP1A1 and CYP1B1 ELISA kits were purchased from MDL Biotech (Beijing, China; n = 6 or 8 for each group). The serum samples were prepared from whole heart blood samples after centrifugation at 3000 × *g* for 10 min. The 10 μl samples and 60 μl dilution buffer were added to the wells followed by incubation at room temperature for 60 min. Then washing the plates and adding zymolytes into the wells, the absorbance was measured on a spectrophotometer at 450 nm. The concentrations were calculated to the amount of standard protein of each sample.

### AHR CALUX cell bioassay [46]

#### Chemicals

TPPU was dissolved in DMSO and provided by EICOSIS at a concentration of 10 mM for use in CALUX bioassay analysis of their agonist and antagonist activity at 10uM final incubation concentration.

#### Bioassay analysis

Recombinant mammalian cells were incubated at the indicated concentration for 24 h, in the absence of reference standard (for agonist activity) or presence of reference standard (antagonist activity), the cells lysed and luciferase activity determined in a microplate luminometer following automatic injection of the reagent (luciferin). The luciferase activity of cells incubated with sample extracts was directly compared to that of cells incubated with negative and positive control chemicals relevant for each bioassay, results expressed as a percent of the maximal induced activity of the respective positive control chemical (after subtraction of background activity) and positive samples confirmed by statistical analysis.

The specific cell bioassays that were run on these two sets of samples included:

1.Human Ah receptor (AHR)-responsive liver cancer cell bioassay for AHR active chemicals (HG2L6.1c1 cells).
2.Mammalian cell lines to detect cytotoxicity (all of the above lines were used for this).

### Cytotoxicity assessment

Cell viability/cytotoxicity was assessed for all experiments using nuclear receptor cell lines using a scaled qualitative visual observation method previously approved by OECD (2016) and ICCVAM (2011) for the VM7Luc4E2 cells that scores viability on a scale of 1 (normal) to 4 (significant loss of viability). Cytotoxicity by this method is identified if cells exhibit any change in normal cell morphology or cell density (the latter resulting from cell death and/or cells detaching from the culture plate). Since no cytotoxicity was observed in any cell line with any chemical or extract treatment they were assigned a score of 1 (Normal Cell Morphology and Cell Density (for details see Table 11–1 in ICCVAM, 2011)).

### Statistical analysis

The data are show as the mean ± standard error of the mean (SEM). Analysis was performed using GraphPad prism software version 7.0. Leven’s test and Shapiro–wilk’s test were used to analyzed the homoscedasticity and normality of each dataset. Comparisons among groups were performed using the one-way analysis of variance (ANOVA) or two-way ANOVA, followed by post hoc Tukey test. For different time-points, two-way repeated ANOVA were performed to compare the difference among groups. If the dataset did not follow a normal distribution or homoscedasticity, nonparametric analyses were introduced to the dataset via Kruskal–Wallis and Mann–Whitney two-sided statistical analyses. In hierarchical cluster analysis, the data were firstly standardized by z scores. Then, hierarchical cluster analysis of SPT was performed using Ward’s method and applying squared Euclidean distance as the distance measure, and mice were classified as anhedonia susceptible or resilient [47]. The P-values of less than 0.05 were considered statistically significant.

## Results

### Altered expression of the sEH protein in selected tissues in sham, anhedonia-susceptible, and anhedonia-resilient mice

**Fig. 1:**
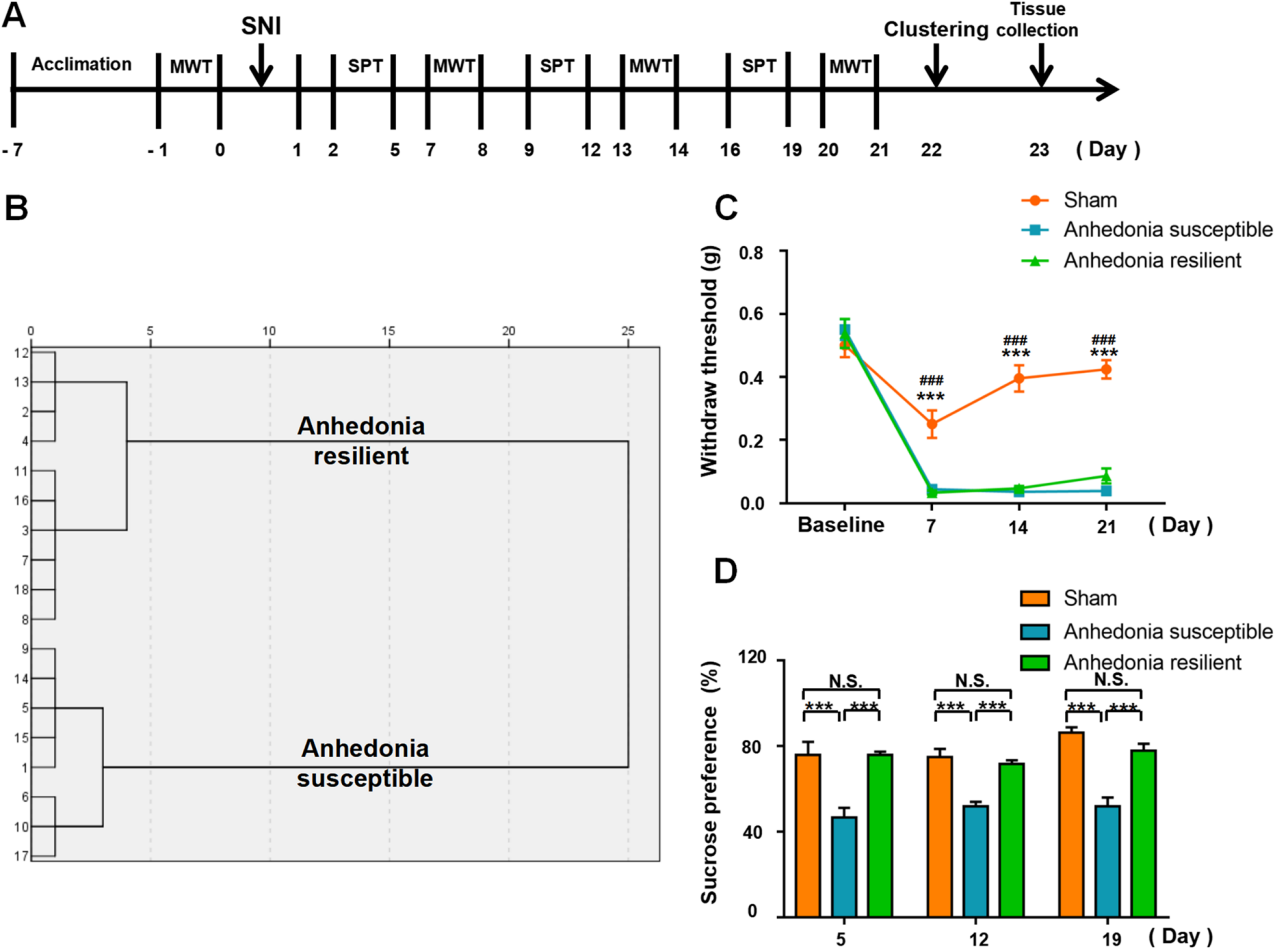
Behavioral results in sham, anhedonia-susceptible, and anhedonia-resilient mice. **A** Schedule of the experiment. SNI surgery was performed after acclimation. MWT was measured on days 7, 14, and 21 after SNI. The SPT was performed on days 5, 12, and 19 after SNI. **B** Dendrogram of the hierarchical clustering analysis. A total of 18 SNI mice were classified into anhedonia-susceptible and anhedonia-resilient groups by a hierarchical cluster analysis of the SPT results. **C** MWT [Time: *F* (3, 21) = 124.2, *P* < 0.001; Group: *F* (2, 14) = 118, *P* < 0.001; Interaction: *F* (6, 42) = 17.12, *P* < 0.001] was measured on days 7, 14, and 21 in the sham, anhedonia-resilient, and anhedonia-susceptible groups after SNI. ^***^P < 0.001, susceptible group vs.. sham group; ^###^*P* < 0.001, resilient group vs.. sham group. **D** SPT [Time: *F* (2, 14) = 1.873, *P* = 0.19; Group: *F* (2, 14) = 49.95, *P* < 0.001; Interaction: *F* (4, 28) = 1.616, *P* = 0.20] was measured in the sham, anhedonia-resilient, and anhedonia-susceptible groups on days 5, 12, and 19 after SNI. ^***^P < 0.001. *MWT* mechanical withdrawal threshold, *SNI* spared nerve injury, *SPT* sucrose preference test

**Fig. 2:**
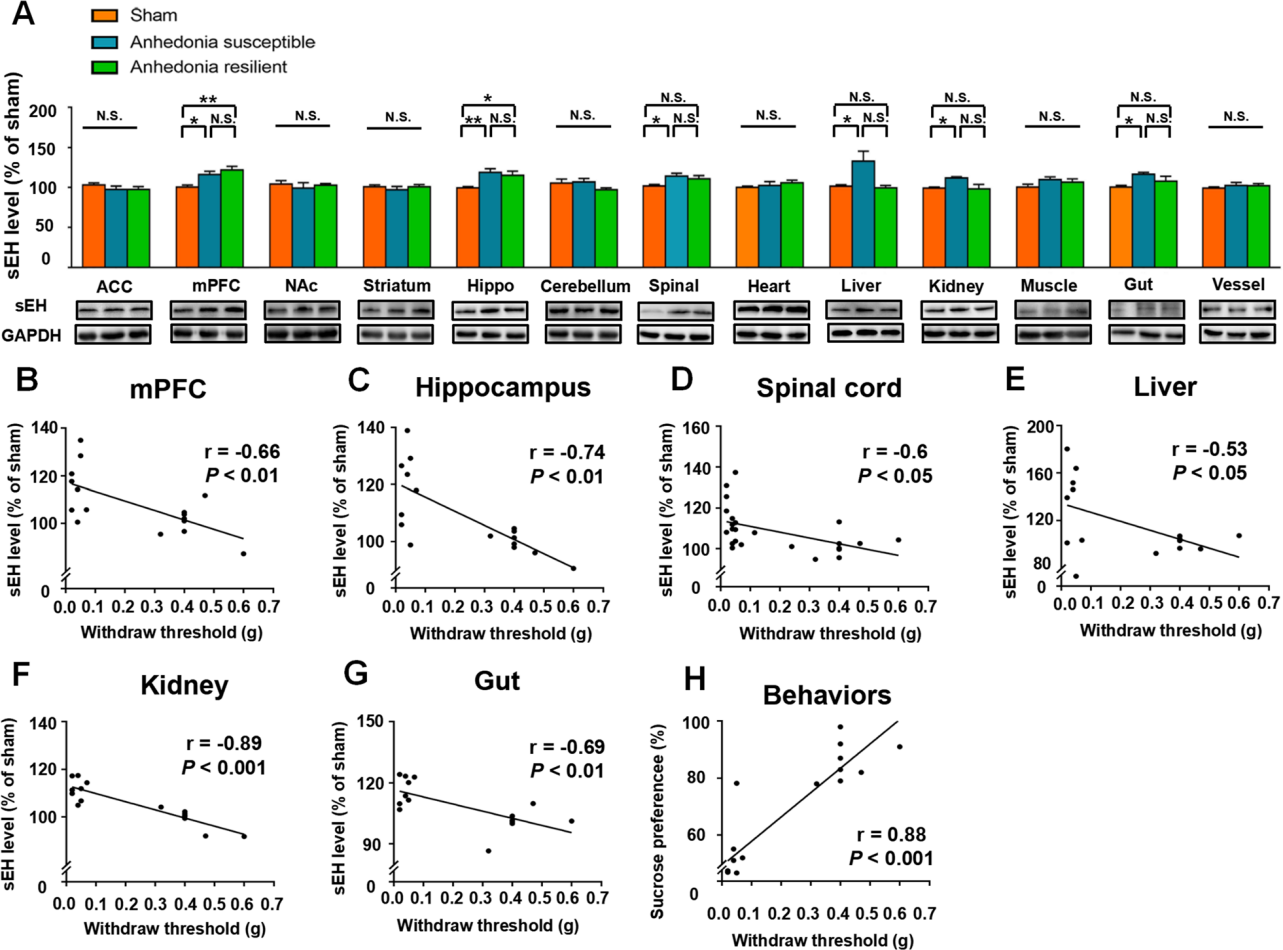
sEH levels in selected tissues from sham, anhedonia-susceptible, and anhedonia-resilient mice. **A** Levels of the sEH protein in selected tissues. ACC [H_2_ = 1.865, *P* = 0.394]; mPFC [*F* (2, 21) = 8.615, *P* < 0.01]; NAc [H_2_ = 0.620, *P* = 0.733]; Striatum [H_2_ = 0.695, *P* = 0.706]; Hippo [*F* (2, 21) = 6.145, *P* < 0.01]; Cerebellum [*F* (2, 21) = 2.75, *P* = 0.087]; Spinal cord [*F* (2, 21) = 3.457, *P* = 0.05]; Heart [H_2_ = 2.945, *P* = 0.229]; Liver [*F* (2, 21) = 6.214, *P* < 0.01]; Kidney [*F* (2, 21) = 4.945, *P* < 0.05]; Muscle [*F* (2, 21) = 1.554, *P* = 0.235]; Gut [H2 = 7.338, *P* < 0.05]; Vessels [*F* (2, 21) = 0.4566, *P* = 0.64]. ^*^P < 0.05; ^**^P < 0.01; ^***^P < 0.001. **B** Correlations between sEH levels and MWT scores in the mPFC (*R*^*2*^ = 0.4302, *P* < 0.01); **C** correlations between sEH levels and MWT scores in the hippocampus (*R*^*2*^ = 0.547, *P* < 0.01); **D** correlations between sEH levels and MWT scores in the spinal cord (*R*^*2*^ = 0.3576, *P* < 0.05); **E** correlations between sEH levels and MWT scores in the liver (*R*^*2*^ = 0.2829, *P* < 0.05); **F** correlations between sEH levels and MWT scores in the kidney (*R*^*2*^ = 0.8007, *P* < 0.001); **G** correlations between sEH levels and MWT scores in the gut (*R*^*2*^ = 0.476, *P* < 0.01); **H** correlations between MWT and SPT scores (*R*^*2*^ = 0.7731, *P* < 0.001). *ACC* anterior cingulate cortex, *mPFC* medial prefrontal cortex, *MWT* mechanical withdrawal threshold, *NAc* nucleus accumbens, NS not significant, *SNI* spared nerve injury, *SPT* sucrose preference test

A total of 18 mice with SNI were classified into anhedonia-susceptible and anhedonia-resilient groups via a hierarchical analysis according to SPT scores (Fig. 1B and Additional file 1: Table S1). The MWT of both anhedonia-susceptible and -resilient mice was dramatically lower than that of the sham group on days 7, 14, and 21 after surgery (Fig. 1C). The SPT scores in anhedonia-susceptible mice were decreased on days 5, 12, and 19 after SNI (Fig. 1D). There were significant changes in the level of sEH in the mPFC, hippocampus, spinal cord, liver, kidney, and gut, but not in the ACC, NAc, striatum, cerebellum, heart, muscle, and blood vessels in anhedonia-susceptible and -resilient mice compared with sham mice (Fig. 2A). These findings suggest that the levels of the sEH protein in selected brain regions might confer susceptibility to chronic neuropathic pain. Next, we investigated the correlations between sEH levels and MWT in selected tissues. Significant negative correlations occurred in tissues such as the mPFC, hippocampus, spinal cord, liver, kidney, and gut (Fig. 2B–G). Furthermore, there was a positive correlation between MWT and SPT scores (Fig. 2H).

### Alleviation of mechanical allodynia and anhedonia-like symptoms in anhedonia-susceptible mice after TPPU treatment

**Fig. 3:**
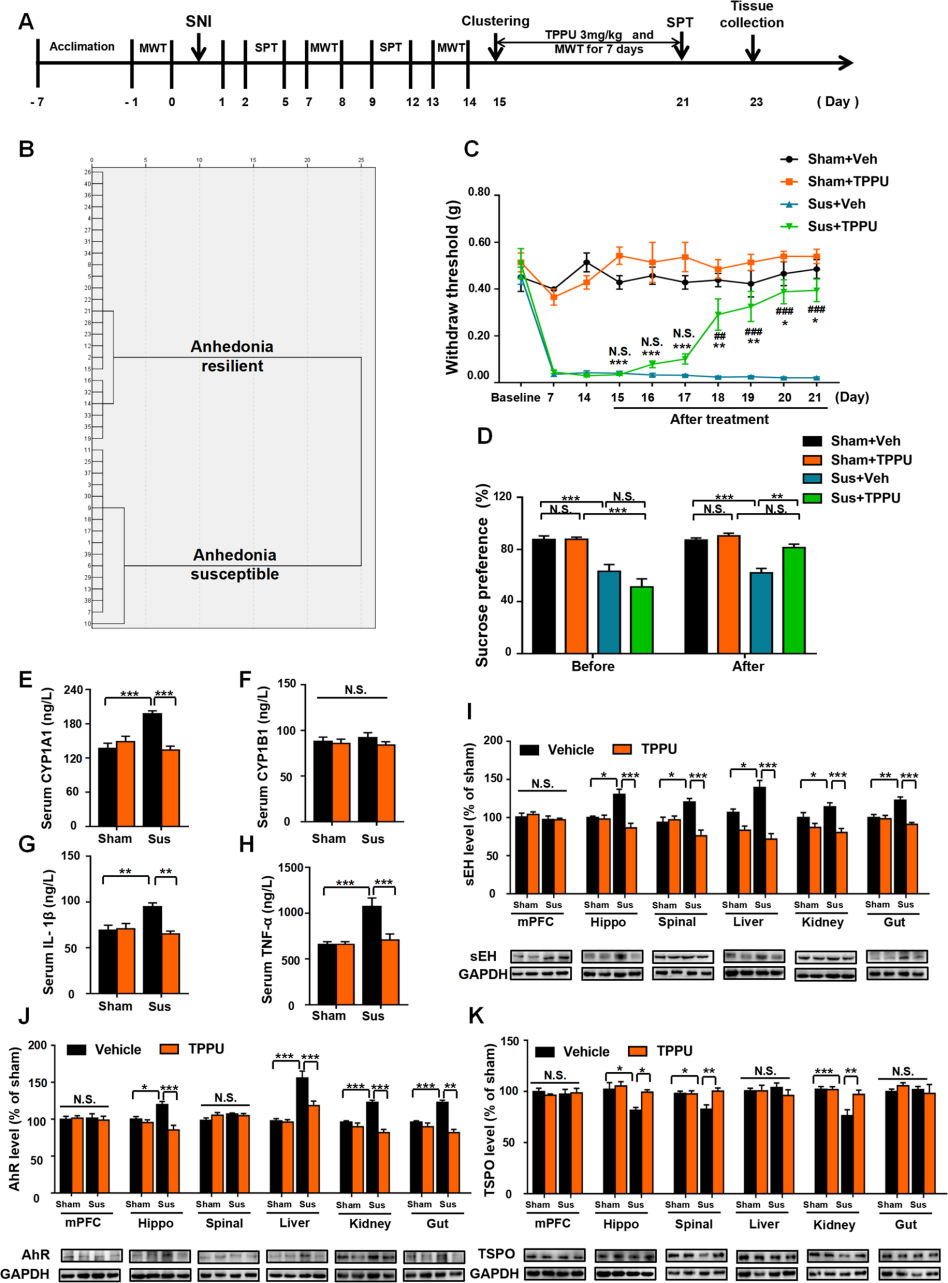
Effects of TPPU on MWT and SPT scores and on the levels of sEH, AHR, and TSPO. **A** Schedule of the experiment. SNI surgery was performed after acclimation. TPPU (3 mg/kg, once daily) was administered orally for 7 consecutive days, starting at day 14 after SNI. MWT was measured on days 7, 14, 15, 16, 17, 18, 19, 20, and 21 after SNI. The SPT was performed on days 5, 12, and 21 after SNI. **B** Dendrogram of the hierarchical clustering analysis. A total of 40 SNI mice were classified into anhedonia-susceptible and anhedonia-resilient groups via a hierarchical cluster analysis of the SPT results. **C** MWT [Time: *F* (9, 63) = 19.33, *P* < 0.001; Group: *F* (3, 21) = 173.5, *P* < 0.001; Interaction: *F* (27, 189) = 7.369, *P* < 0.001] was measured on days 7, 14, 15, 16, 17, 18, 19, 20 and 21 in the Sham + Veh, Sham + TPPU, Sus + Veh, and Sus + TPPU groups after SNI. ^***^P < 0.001, Sham + Veh group vs. Sus + Veh group; ^###^*P* < 0.001, Sus + Veh group vs. Sus + TPPU group. **D** SPT [Time: *F* (1, 56) = 10.34, *P* < 0.01; Group: *F* (3, 56) = 33.17, *P* < 0.001; Interaction: *F* (3, 56) = 8.744, *P* < 0.001] was performed in the Sham + Veh, Sham + TPPU, Sus + Veh, and Sus + TPPU groups on days 12 and 21 after SNI. ^**^P < 0.01, ^***^P < 0.001. **E** CYP1A1 level in the serum [TPPU: *F* (1, 20) = 10.73, *P* < 0.01; Group: *F* (1, 20) = 8.407, *P* < 0.01; Interaction: *F* (1, 20) = 22.69, *P* < 0.001]. **F** CYP1B1 level in the serum [TPPU: *F* (1, 20) = 1.241, *P* = 0.28; Group: *F* (1, 20) = 0.06, *P* = 0.8; Interaction: *F* (1, 20) = 0.436, *P* = 0.52]. **G** IL-1β level in the serum [TPPU: *F* (1, 20) = 8.976, *P* < 0.01; Group: *F* (1, 20) = 4.557, *P* < 0.05; Interaction: *F* (1, 20) = 11.16, *P* < 0.01]. **(H)** TNF-α level in the serum [TPPU: *F* (1, 20) = 55.45, *P* < 0.001; Group: *F* (1, 20) = 88.65, *P* < 0.001; Interaction: *F* (1, 20) = 55.45, *P* < 0.001]. **I** Effects of TPPU on sEH expression. mPFC [TPPU: *F* (1, 28) = 0.1376, *P* = 0.71; Group: *F* (1, 28) = 1.615, *P* = 0.21; Interaction: *F* (1, 28) = 0.2046, *P* = 0.65]; hippocampus [TPPU: *F* (1, 28) = 18.62, *P* < 0.001; Group: *F* (1, 28) = 3.055, *P* = 0.09; Interaction: *F* (1, 28) = 15.77, *P* < 0.001]; spinal cord [TPPU: *F* (1, 28) = 11.33, *P* < 0.001; Group: *F* (1, 28) = 0.1966, *P* = 0.67; Interaction: *F* (1, 28) = 15.28, *P* < 0.001]; liver [TPPU: *F* (1, 28) = 44.43, *P* < 0.001; Group: *F* (1, 28) = 2.362, *P* = 0.14; Interaction: *F* (1, 28) = 10.38, *P* < 0.01]; kidney [TPPU: *F* (1, 28) = 51.25, *P* < 0.001; Group: *F* (1, 28) = 1.287, *P* = 0.26; Interaction: *F* (1, 28) = 9.837, *P* < 0.01]; gut [TPPU: *F* (1, 28) = 18.49, *P* < 0.001; Group: *F* (1, 28) = 3.879, *P* = 0.06; Interaction: *F* (1, 28) = 15.33, *P* < 0.001]. ^*^P < 0.05, ^**^P < 0.01, ^***^P < 0.001. **J** Effects of TPPU on AHR expression. mPFC [TPPU: *F* (1, 28) = 0.0119, *P* = 0.91; Group: *F* (1, 28) = 0.016, *P* = 0.9; Interaction: *F* (1, 28) = 0.1949, *P* = 0.66]; hippocampus [TPPU: *F* (1, 28) = 18.04, *P* < 0.001; Group: *F* (1, 28) = 1.015, *P* = 0.32; Interaction: *F* (1, 28) = 10.42, *P* < 0.01]; spinal cord [TPPU: *F* (1, 28) = 0.6641, *P* = 0.42; Group: *F* (1, 28) = 1.8, *P* = 0.19; Interaction: *F* (1, 28) = 2.695, *P* = 0.11]; liver [TPPU: *F* (1, 28) = 10.73, *P* < 0.01; Group: *F* (1, 28) = 44.97, *P* < 0.001; Interaction: *F* (1, 28) = 8.917, *P* < 0.01]; kidney [TPPU: *F* (1, 28) = 39.57, *P* < 0.001; Group: *F* (1, 28) = 5.957, *P* < 0.05; Interaction: *F* (1, 28) = 21.38, *P* < 0.001]; gut [TPPU: *F* (1, 28) = 5.764, *P* < 0.05; Group: *F* (1, 28) = 59.08, *P* < 0.001; Interaction: *F* (1, 28) = 6.479, *P* < 0.05]. Data are reported as the mean ± SEM (n = 8). ^*^P < 0.05, ^**^P < 0.01, ^***^P < 0.001. **K** Effects of TPPU on TSPO expression. mPFC [TPPU: *F* (1, 28) = 0.0844, *P* = 0.77; Group: *F* (1, 28) = 0.0057, *P* = 0.94; Interaction: *F* (1, 28) = 0.4106, *P* = 0.53]; hippocampus [TPPU: *F* (1, 28) = 6.114, *P* < 0.05; Group: *F* (1, 28) = 9.662, *P* < 0.01; Interaction: *F* (1, 28) = 2.828, *P* = 0.1]; spinal cord [TPPU: *F* (1, 28) = 6.981, *P* < 0.05; Group: *F* (1, 28) = 3.933, *P* = 0.06; Interaction: *F* (1, 28) = 7.76, *P* < 0.01]; liver [TPPU: *F* (1, 28) = 0.7191, *P* = 0.4; Group: *F* (1, 28) = 0.015, *P* = 0.9; Interaction: *F* (1, 28) = 0.6986, *P* = 0.41]; kidney [TPPU: *F* (1, 28) = 6.278, *P* < 0.05; Group: *F* (1, 28) = 14.19, *P* < 0.001; Interaction: *F* (1, 28) = 6.607, *P* < 0.05]; gut [TPPU: *F* (1, 28) = 0.3, *P* = 0.86; Group: *F* (1, 28) = 0.3095, *P* = 0.58; Interaction: *F* (1, 28) = 0.9039, *P* = 0.34]. Data are reported as the mean ± SEM (n = 8). ^*^P < 0.05, ^**^P < 0.01, ^***^P < 0.001. *AHR* aryl hydrocarbon receptor, *CYP1A1* cytochrome P450, family 1, subfamily A, polypeptide 1, *CYP1B1* cytochrome P450, family 1, subfamily B, polypeptide 1, *sEH* soluble epoxide hydrolase, *mPFC* medial prefrontal cortex, *NS* not significant, *SNI* spared nerve injury, *Sus* susceptible, *TPPU* 1-trifluoromethoxyphenyl-3-(1-propionylpiperidin-4-yl) urea, *TSPO* translocator protein, *Veh* vehicle

Sixteen anhedonia-susceptible mice were selected after hierarchical cluster analysis of SPT scores (Fig. 3A, 3B and Additional file 1: Table S2). To detect the effects of TPPU on mechanical allodynia and anhedonia, we treated the sham group and anhedonia-susceptible mice daily with vehicle or TPPU (3 mg/kg) for 7 consecutive days. TPPU improved the decreased SPT scores and increased the decreased scores of MWT in anhedonia-susceptible mice (Fig. 3C, D). After administration of TPPU, the levels of sEH were decreased significantly in the hippocampus, spinal cord, liver, kidney, and gut, but not in the mPFC of anhedonia-susceptible mice compared to the sham group (Fig. 3I). Intriguingly, two contradictory trends emerged regarding TSPO and AHR signaling. The AHR levels were increased in the hippocampus, liver, kidney, and gut but not in the mPFC and spinal cord of anhedonia-susceptible mice. In contrast, the TSPO levels were decreased in the hippocampus, spinal cord, and kidney in the anhedonia-susceptible group. Finally, TPPU reversed these changes (Fig. 3J–K). Our results indicated a similar tendency between the levels of sEH and AHR, but an opposite trend between the levels of sEH and TSPO. In addition, we examined the levels of inflammatory cytokines and showed that the serum concentrations of CYP1A1, IL-1β, and TNF-α were increased in anhedonia-susceptible mice. In contrast, a decrease in the serum levels of CYP1A1, IL-1β, and TNF-α occurred after TPPU treatment (Fig. 3E–H). These data suggest that TPPU exerts its beneficial effects via the inhibition of AHR signaling and the activation of TSPO signaling.

### An AHR agonist attenuated the antidepressant-like effects of TPPU

**Fig. 4:**
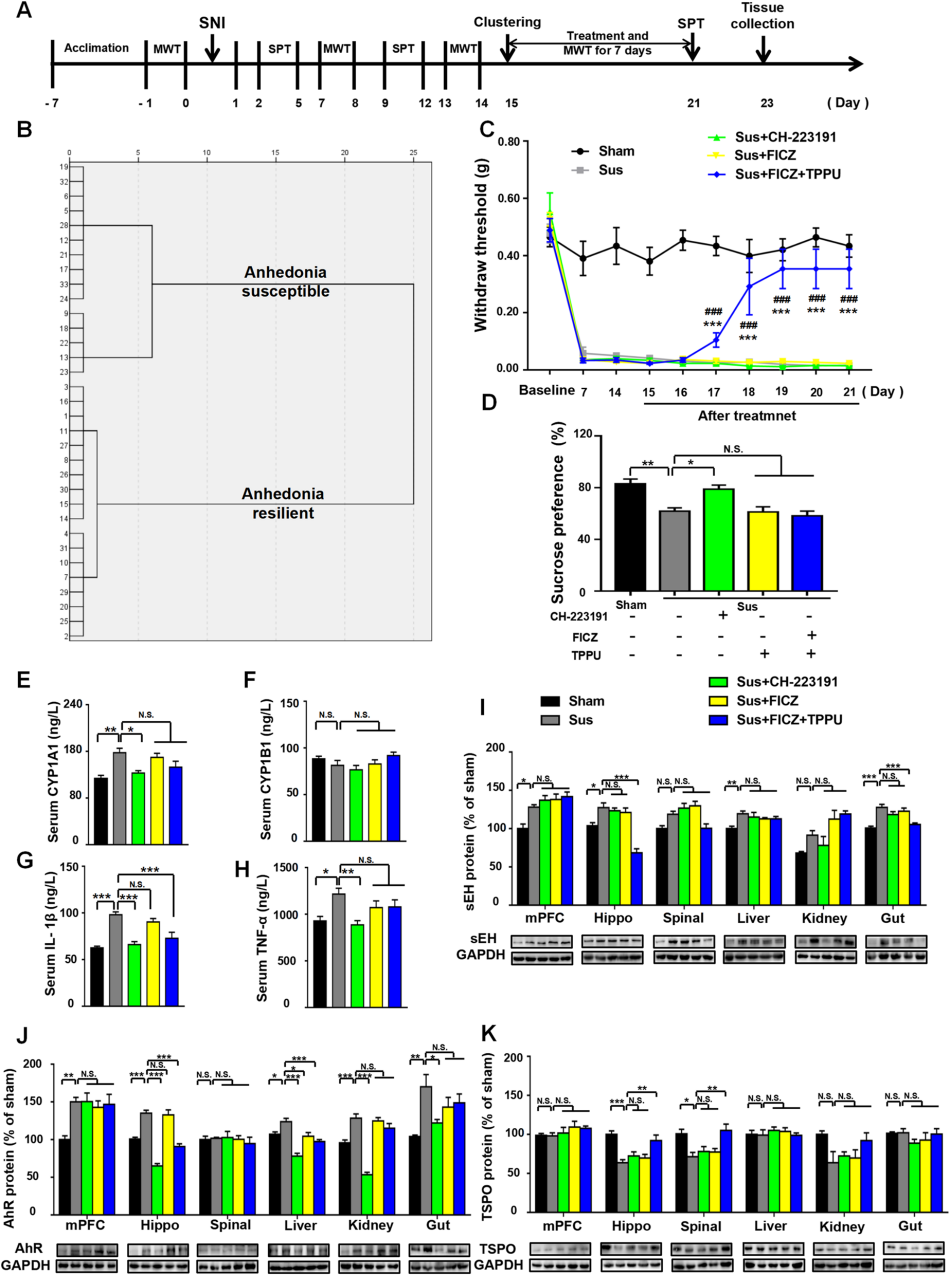
An AHR agonist attenuated the antidepressant effects, but not the analgesic effects of TPPU. **A** Schedule of the experiment. SNI surgery was performed after acclimation. CH-223191 (10 mg/kg, once daily) and TPPU (3 mg/kg, once daily) were administered orally for 7 consecutive days, starting at 14 after SNI. FICZ (100 µg/mg) was administered orally on day 14. MWT was measured on days 7, 14, 15, 16, 17, 18, 19, 20, and 21 after SNI. The SPT was performed on days 5, 12, and 21 after SNI. **B** Dendrogram of the hierarchical clustering analysis. A total of 33 SNI mice were classified into anhedonia-susceptible and anhedonia-resilient groups based on SPT scores. **C** MWT [Time: *F* (9, 45) = 61.2, *P* < 0.001; Group: *F* (4, 20) = 128.8, *P* < 0.001; Interaction: *F* (36, 180) = 8.887, *P* < 0.001] was measured on days 7, 14, 15, 16, 17, 18, 19, 20, and 21 after SNI in the Sham, Sus, Sus + CH-223191, Sus + FICZ, and Sus + FICZ + TPPU groups. ^***^P < 0.001, Sus group vs. Sus + FICZ + TPPU group; ^###^*P* < 0.001, Sus + FICZ group vs. Sus + FICZ + TPPU group. **D** The SPT [*F*(4, 25) = 10.86, *P* < 0.001] was performed in the sham, Sus, Sus + CH-223191, Sus + FICZ, and Sus + FICZ + TPPU groups on day 21 after SNI. *P < 0.05, **P < 0.01. **E** CYP1A1 level in the serum [*F* (4, 25) = 6.211, *P* < 0.01].**F** CYP1B1 level in the serum [*F* (4, 25) = 1.964, *P* = 0.13]. **G** IL-1β level in the serum [*F* (4, 25) = 15.85, *P* < 0.01]. **H** TNF-α level in the serum [*F* (4, 25) = 4.449, *P* < 0.01]. **I** Effects of TPPU on sEH expression. mPFC [*F* (4, 25) = 8.255, *P* < 0.001]; hippocampus [*F* (4, 25) = 21.93, *P* < 0.001]; spinal cord [*F* (4, 25) = 7.137, *P* < 0.001]; liver [*F* (4, 25) = 3.81, *P* < 0.05]; kidney [*F* (4, 25) = 7.236, *P* < 0.001]; gut [*F* (4, 25) = 12.33, *P* < 0.001]. *P < 0.05, **P < 0.01, ***P < 0.001. **J** Effects of TPPU on AHR expression. mPFC [*F* (4, 25) = 5.272, *P* < 0.01]; hippocampus [*F* (4, 25) = 48.4, *P* < 0.001]; spinal cord [*F* (4, 25) = 0.2683, *P* = 0.90]; liver [*F* (4, 25) = 18.69, *P* < 0.001]; kidney [*F* (4, 25) = 41.8, *P* < 0.001]; gut [*F* (4, 25) = 5.395, *P* < 0.01]. ^*^P < 0.05, ^**^P < 0.01, ^***^P < 0.001. **K** Effects of TPPU on TSPO expression. mPFC [*F* (4, 25) = 0.9551, *P* = 0.45]; hippocampus [*F* (4, 25) = 8.753, *P* < 0.001]; spinal cord [*F* (4, 25) = 6.161, *P* < 0.01]; liver [*F* (4, 25) = 0.3481, *P* = 0.84]; kidney [*F* (4, 25) = 2.627, *P* = 0.059]; gut [*F* (4, 25) = 0.9321, *P* = 0.46]. ^*^P < 0.05, ^**^P < 0.01, ^***^P < 0.001. *AHR* aryl hydrocarbon receptor, *CYP1A1* cytochrome P450, family 1, subfamily A, polypeptide 1, *CYP1B1* cytochrome P450, family 1, subfamily B, polypeptide 1, *FICZ* 6-formylindolo[3,2-b]carbazole, *sEH* soluble epoxide hydrolase, *mPFC* medial prefrontal cortex, *NS* not significant, *SNI* spared nerve injury, *Sus* susceptible, *TPPU* 1-trifluoromethoxyphenyl-3-(1-propionylpiperidin-4-yl) urea, *TSPO* translocator protein

Mice with SNI-induced anhedonia were selected by hierarchical cluster analysis of SPT scores (Fig. 4A, B, Additional file 1: Fig. S1, and Additional file 1: Tables S3 and S4). We administered an AHR agonist (FICZ, 100 μg/kg), an AHR antagonist (CH-223191, 10 mg/kg), and TPPU (3 mg/kg) to anhedonia-susceptible mice. Pain relief behaviors occurred after treating anhedonia-susceptible mice with TPPU and FICZ (Fig. 3C). More importantly, CH-223191 alone restored the decreased SPT scores in anhedonia-susceptible mice (Fig. 3D). CH-223191 exerted pharmacological benefits regarding inhibited levels of CYP1A1, IL-1β, and TNF-α, but not of CYP1B1. The serum IL-1β level was decreased after the combined utilization of TPPU and FICZ in anhedonia-susceptible mice (Fig. 3E–H). In the hippocampus and gut, the levels of the sEH protein were decreased in anhedonia-susceptible mice that received FICZ and TPPU (Fig. 3I). In addition, CH-223191 decreased the sEH level in the hippocampus, liver, kidney, and gut. Moreover, the combined use of FICZ and TPPU also decreased the AHR levels in anhedonia-susceptible mice (Fig. 3J). In addition, we also found TPPU is neither an agonist nor an antagonist in the AHR CALUX cell bioassay and that no cytotoxicity was observed at a concentration of 10 uM in the incubation (Table. 1**)**.

**Table 1:** Stimulation/inhibition of AH receptor (AHR)-responsive luciferase reporter gene activity in H1L6.1c3 cells by sample extracts

Chemical treatment	Agonist activity	Antagonist activity
TCDD	100 ± 29.14	100 ± 28.68
TPPU	− 0.70 ± 2.66	85.18 ± 31.23

Results are expressed as the mean ± SD of triplicate incubations of each sample and an asterisk (*) indicates those values significantly greater than the background activity in the DMSO blank at P < 0.05 as determined by the Students t-test. Results in this table demonstrate that no chemical significantly induced AHR-dependent reporter gene activity above background activity or antagonized induction by the reference standard, TCDD. (Table 1)

Conversely, the levels of the TSPO protein were increased in the hippocampus and spinal cord in anhedonia-susceptible mice after the administration of FICZ and TPPU (Fig. 3K). These results indicate that the AHR antagonist exert the antidepressant-like effect similar to TPPU, whereas no change in its analgesic effect appeared in anhedonia-susceptible mice. These findings suggest that the downregulation of AHR may improve the SPT scores in anhedonia-susceptible mice.

### Both the antidepressant-like and analgesic effects of TPPU were attenuated after treatment with a TSPO antagonist

**Fig. 5:**
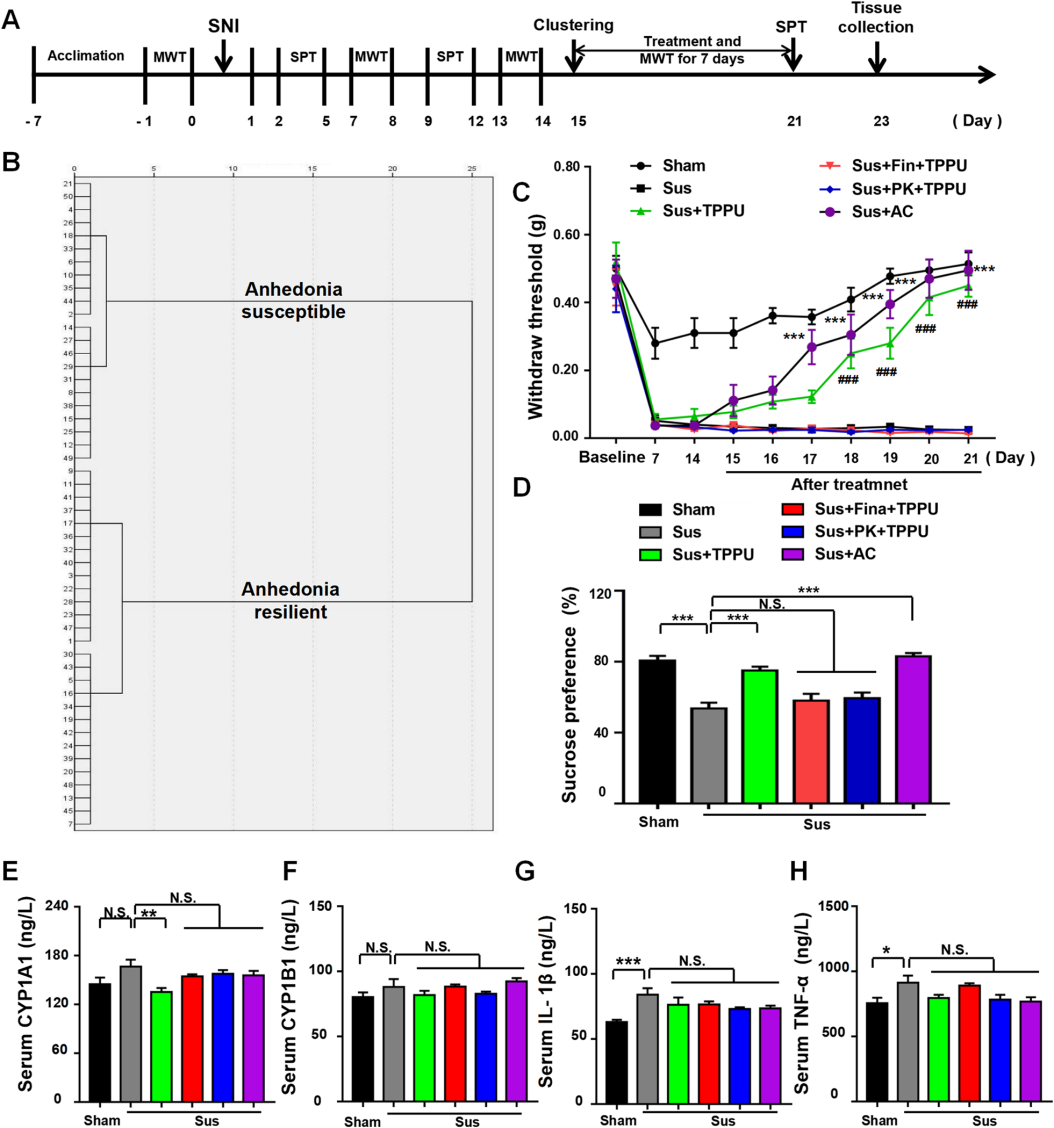
A TSPO antagonist attenuated the antidepressant and analgesic effects of TPPU. **A** Schedule of the experiment. SNI surgery was performed on day 0 after acclimation. Finasteride (10 mg/kg, once daily) or PK-11195 (3 mg/kg, once daily) was administered orally together with TPPU (3 mg/kg, once daily) for 7 consecutive days, starting at day 14 after SNI. AC-5216 (5 mg/mg) was administered orally from day 14 to day 21. MWT was measured on days 7, 14, 15, 16, 17, 18, 19, 20, and 21 after SNI. The SPT was performed on days 5, 12, and 21 after SNI. **B** Dendrogram of the hierarchical clustering analysis. A total of 36 SNI mice were classified into anhedonia-susceptible and anhedonia-resilient groups based on SPT scores. **C** MWT [Time: *F* (9, 63) = 73.44, *P* < 0.001; Group: *F* (5, 35) = 156.5, *P* < 0.001; Interaction: *F* (45, 315) = 9.604, *P* < 0.001] was measured on days 7, 14, 15, 16, 17, 18, 19, 20, and 21 after SNI in the Sham, Sus, Sus + TPPU, Sus + Fina + TPPU, Sus + PK + TPPU, and Sus + AC groups. ^***^P < 0.001, Sus group vs. Sus + AC group; ^###^*P* < 0.001, Sus group vs. Sus + TPPU group. **D** The SPT [*F*(5, 42) = 18.98, *P* < 0.001] was performed on day 21 after SNI in the Sham, Sus, Sus + TPPU, Sus + Fina + TPPU, Sus + PK + TPPU, and Sus + AC groups. ^***^P < 0.001. **E** CYP1A1 level in the serum [*F* (5, 42) = 3.299, *P* < 0.05]. **F** CYP1B1 level in the serum [*F* (5, 42) = 1.825, *P* = 0.13]. **G** IL-1β level in the serum [*F* (5, 42) = 4.101; *P* < 0.01]. **H** TNF-α level in the serum [*F* (5, 42) = 3.41; *P* < 0.05]. ^*^P < 0.05, ^**^P < 0.01, ^***^P < 0.001. *AC* AC-5216, *AHR* aryl hydrocarbon receptor, *CYP1A1* cytochrome P450, family 1, subfamily A, polypeptide 1, *CYP1B1* cytochrome P450, family 1, subfamily B, polypeptide 1; Fina, finasteride, *NS* not significant, *SNI* spared nerve injury; Sus, susceptible, *TPPU* 1-trifluoromethoxyphenyl-3-(1-propionylpiperidin-4-yl) urea, *PK* PK-11195

Anhedonia-susceptible mice were selected and treated with a TSPO agonist (AC5216), TSPO antagonists (PK-11195 and Finasteride), and TPPU (Fig. 5A, B, Additional file 1: Fig. S2, and Additional file 1: Tables S5 and S6). AC5216 exerted antidepressant-like and analgesic effects similar to those of TPPU in anhedonia-susceptible mice (Fig. 5C and D). Furthermore, TPPU reduced serum CYP1A1 in anhedonia-susceptible mice (Fig. 5E–H). Therefore, the activation of TSPO signaling by TPPU may have effective antidepressant-like and analgesic effects in anhedonia-susceptible mice.

## Discussion

Despite suffering from similar nociceptive damage, only some of the SNI mice exhibited the anhedonia phenotype. The tissue levels of sEH were increased in the mPFC, hippocampus, spinal cord, liver, kidney, and gut, but not in the ACC, NAc, striatum, cerebellum, heart, muscle, and vessels in anhedonia-susceptible mice compared with sham or anhedonia-resilient mice. We also found that the sEH level in selected tissues was negatively correlated with MWT scores, whereas the scores of the SPT positively correlated with the scores of the MWT. This suggests that sEH levels are closely related to neuropathic pain and that the results of the behavioral tests also changed after the onset of pain. Furthermore, TPPU not only reduced sEH protein levels, but also improved the anhedonia and hyperalgesia behaviors. In addition, we found that TPPU reduced the serum levels of inflammatory cytokines and CYP1A1. More importantly, the levels of two other proteins, AHR and TSPO, were also altered in selected tissues. Surprisingly, pretreatment with an AHR agonist attenuated the antidepressant-like but not the analgesic effect of TPPU. Furthermore, a TSPO agonist exerted a TPPU-like antidepressant effect.

Both chronic pain and depression seriously affect the patients’ quality of life affected by them [48, 49]. However, they are frequently encountered clinically, thus increasing the morbidity and recurrence rates and rendering treatment more difficult [50]. According to epidemiological studies, chronic pain in patients treated for depression is as high as 51.8–59.1%; moreover, depression in patients with chronic pain reportedly ranges from 18 to 85% [51–54]. In preclinical studies, persistent pain frequently induced depression-like behaviors [55]. Furthermore, chronic inflammatory pain and neuropathic pain often lead to hyperalgesia in animals. Some animals simultaneously exhibit anhedonia and behavioral despair [41, 56]. In this study, a hierarchical cluster analysis classified SNI mice into two clusters: one group (approximately 44%, anhedonia-like symptoms) had decreased scores in the SPT, whereas the other group (approximately 56%, without anhedonia-like symptoms) exhibited sucrose preference, similar to that observed in sham-operated mice and consistent with our previous report [57].

We found increased levels of sEH in the mPFC, hippocampus, spinal cord, liver, kidney, and gut. The liver, kidney, and intestine are important organs involved in immune response and the release of inflammatory cytokines. The mPFC, hippocampus, and spinal cord play an integral role in the pathogenesis of depression and pain [41, 58–61]. Recent preclinical studies showed that the expression levels of sEH in the mPFC, striatum, and hippocampus were higher in mice with depression-like behaviors than they were in control mice. Moreover, *sEH*-KO mice showed resilience to repeated social defeat stress [21]. In addition, sEH inhibitors also significantly relieved hyperalgesia in rats with chronic pain [62]. Accordingly, in the present study, we found that TPPU inhibited the expression of sEH and decreased the release of inflammatory cytokines, thus exerting analgesic and antidepressant effects.

AHR controls the conversion of PUFAs to EETs by regulating the transcription of the *CYP* family of genes [63]. It was interesting to detect an increased serum level of CYP1A1, but not CYP1B1 in anhedonia-susceptible mice. This discrepancy may result from the different spectra of eicosanoids obtained from ARA in the CYP family. CYP1A1 tends to produce HETEs, whereas CYP1B1 favors EETs [64]. Together with anti-inflammatory EETs, PUFAs are also involved in the biosynthesis of pro-inflammatory mediators through COX and LOX pathways and CYP hydroxylation [65]. Thus, sEH amplified the inflammatory reaction by deactivating EETs and other EpEAs. Several studies suggested AHR overactivation in the hippocampus contributes to neuroinflammatory diseases [25]. Similarly, we found significantly elevated AHR expression in the hippocampus of anhedonia-susceptible mice, which was consistent with changes in sEH. However, there were no changes in the spinal cord. Moreover, TPPU failed to exert an antidepressant-like effect after pretreatment with an AHR agonist. This suggests that AHR signaling is involved exclusively in the occurrence of depression-like symptoms and explains why AHR expression in the spinal cord was not different between the anhedonia-susceptible mice and the sham group.

In addition to synergistically promoting depressive symptoms with AHR, sEH can also inhibit normal TSPO function by inducing the co-occurrence of pain and depression. EETs promoted the transport of cholesterol in mitochondria via TSPO, leading to the production of neurosteroids [66]. In turn, neurosteroids can affect neuron survival and differentiation by regulating neurotrophic factors and anti-inflammatory cytokines [67, 68]. The activation of TSPO in the spinal cord relieves neuropathic and inflammatory pain [69]. It has been reported that steroidogenic actute regulatory protein (StARD1) and TSPO cooperatively exert antihyperalgesic effect by EETs or sEHIs [70]. Furthermore, TSPO overexpression in the hippocampus attenuated depression-like behaviors associated with increased neurosteroid synthesis [71]. In the present study, we found decreased expression of TSPO in the hippocampus and spinal cord in anhedonia-susceptible mice, which might stem from the increased activity of sEH. More importantly, the analgesic and antidepressant effects of the TSPO agonist did not reduce the peripheral levels of inflammatory cytokines, which suggests that the primary therapeutic target of TSPO in the brain. Previous studies showed that the low concentration of neurosteroids produced by the neuronal autocrine system quickly and efficiently exerted pharmacological effects locally [72, 73]. We need futher devote our effort to explore the other potential agents for chronic pain and depression comorbidity.

## Conclusions

**Fig. 6:**
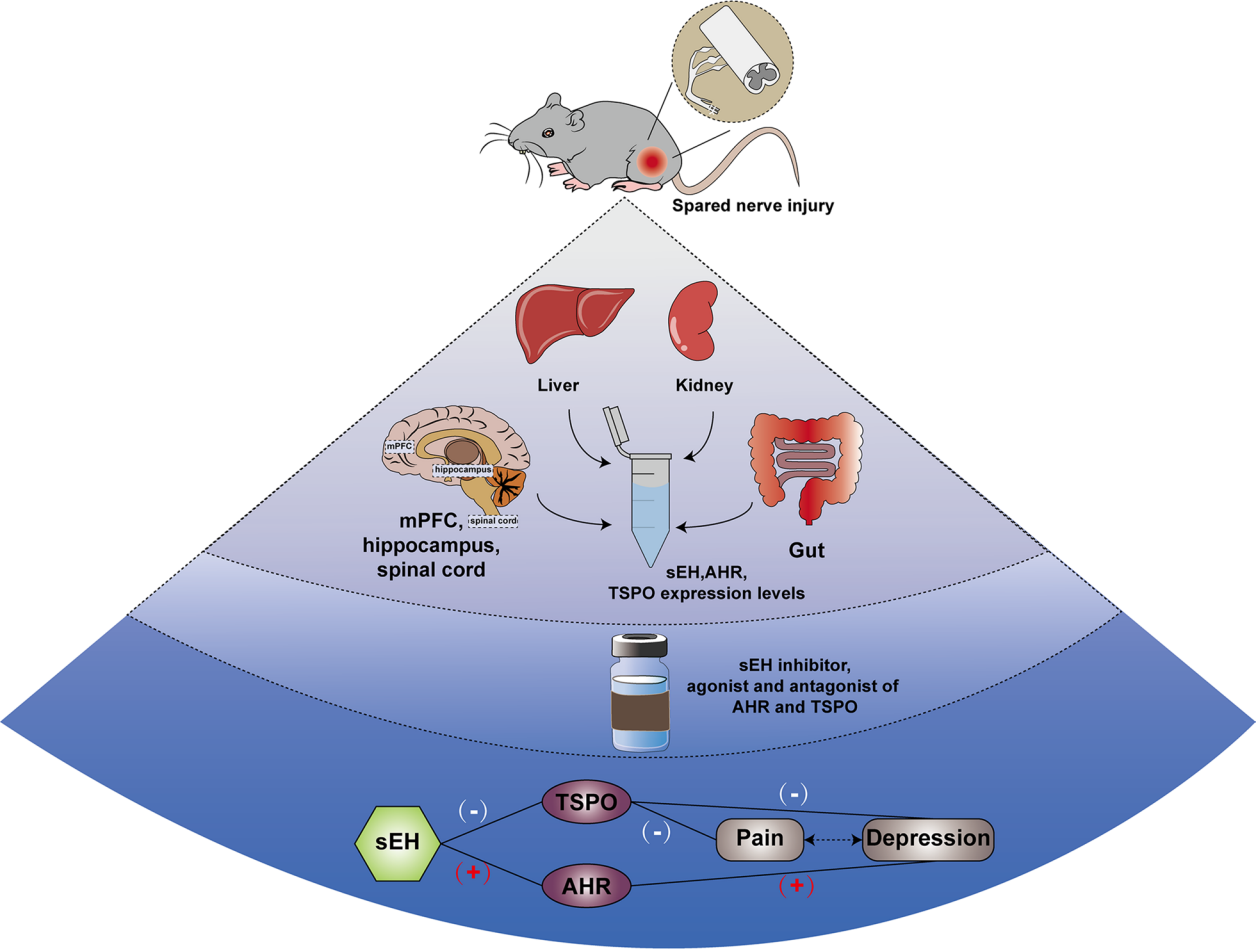
The soluble epoxide hydrolase inhibitor TPPU improves comorbidity of chronic pain and depression via AHR and TSPO signaling. Spared nerve ingury (SNI) was used for the rodent model of pain-depression comorbidity. sEH inhibitor exerted antidepressant-like and analgesic effects in two ways: (1) inhibiting AHR signaling to function as antidepressant; and (2) improving depressive-like and hyperalgesic behaviors by increasing the TSPO protein levels

Our present study results suggest that elevated levels of sEH play an important role in neuropathic pain with anhedonia(Fig. 6). Moreover, TPPU may alleviate the anhedonia and pain behaviors in two ways: (1) reducing the release of peripheral inflammatory factors via the inhibition of AHR signaling, to function as an antidepressant; and (2) improving depressive-like and hyperalgesic behaviors by promoting the synthesis of neurosteroids involved in TSPO signaling. Therefore, these findings provide novel therapeutic targets for chronic pain and depression comorbidity.

## Supplementary Information

**Additional file 1**: **Figure S1**. Dendrogram of the hierarchical clustering analysis. A total of 27 SNI mice were classified into anhedonia-susceptible and anhedonia-resilient groups by a hierarchical cluster analysis of the SPT results. SNI, spared nerve injury; SPT, sucrose preference test. **Figure S2**. Dendrogram of the hierarchical clustering analysis. A total of 52 SNI mice were classified into anhedonia-susceptible and anhedonia-resilient groups by a hierarchical cluster analysis of the SPT results. SNI, spared nerve injury; SPT, sucrose preference test. **Table** **S1**. (Additional material for figure 1B). **Table** **S2**. (Additional material for figure 3B). **Table** **S3**. (Additional material for figure 4B). **Table** **S4**. (Additional material for figure S1). **Table** **S5**. (Additional material for figure 5B). **Table** **S6**. (Additional material for S2).

## Data Availability

The datasets used during this study are available from the corresponding author on reasonable request.

## Abbreviations

sEH: Soluble epoxide hydrolase
SPT: Sucrose preference test
SNI: Spared nerve injury
mPFC: Medial prefrontal cortex
IL-1β: Interleukin 1β
TNF-α: Tumor necrosis factor α
MWT: Mechanical withdrawal threshold
AHR: Aryl hydrocarbon receptor
TSPO: Translocator protein
ARA: Arachidonic acid
CYP450s: Cytochrome P450s
EpFAs: Epoxy fatty acids
EETs: Epoxyeicosatrienoic acids
PUFAs: Polyunsaturated fatty acids
EPA: Eicosapentaenoic acid
DHA: Docosahexaenoic acid
EEQs: Epoxyeicosatetraenoic acids
EDPs: Epoxydocosapentaenoic acids
EpFAs: Epoxy-fatty acids
VCAM-1: Vascular cell adhesion molecule 1
ICAM-1: Intercellular adhesion molecule 1
E-selectin: Endothelial cell-selective adhesion molecule
NF-κB: Nuclear factor κB
DHETs: Dihydroxyeicosatrienoic acids
sEHIs: SEH inhibitors
LPS: Lipopolysaccharide
ARNT: AHR nuclear translocator
HETEs: Hydroxyeicosatetraenoic acids
