# Supplementary material for: The soluble epoxide hydrolase inhibitor TPPU improves comorbidity of chronic pain and depression via the AHR and TSPO signaling
Source: J Transl Med. 2023 Feb 2;21:71. doi: 10.1186/s12967-023-03917-x (PMC9896784; doi:10.1186/s12967-023-03917-x)
Supplement: Supplementary file 1 — Additional file 1: Figure S1. Dendrogram of the hierarchical clustering analysis. A total of 27 SNI mice were classified into anhedonia-susceptible and anhedonia-resilient groups by a hierarchical cluster analysis of the SPT results. SNI, spared nerve injury; SPT, sucrose preference test. Figure S2. Dendrogram of the hierarchical clustering analysis. A total of 52 SNI mice were classified into anhedonia-susceptible and anhedonia-resilient groups by a hierarchical cluster analysis of the SPT results. SNI, spared nerve injury; SPT, sucrose preference test. Table S1. (Additional material for figure 1B). Table S2. (Additional material for figure 3B). Table S3. (Additional material for figure 4B). Table S4. (Additional material for figure S1). Table S5. (Additional material for figure 5B). Table S6. (Additional material for S2). [file 12967_2023_3917_MOESM1_ESM.docx]

***Supplementary material***

**The soluble epoxide hydrolase inhibitor TPPU improves comorbidity of chronic pain and depression via the AHR and TSPO signaling**

Ailin Luo1,#, Zifeng Wu2,#, Shan Li1,#, [Cindy B. McReynolds](https://pubmed.ncbi.nlm.nih.gov/?sort=date&size=50&term=McReynolds+CB&cauthor_id=35312372)4, Di Wang2, Hanyu Liu2, Chaoli Huang2,3, Teng He2, Xinying Zhang2, Yuanyuan Wang2, Cunming Liu2, Bruce D. Hammock4,*, Kenji Hashimoto5,*, Chun Yang2,*

1Department of Anesthesiology, Tongji Hospital, Tongji Medical College, Huazhong University of Science and Technology, Wuhan 430030, China

2Department of Anesthesiology and Perioperative Medicine, The First Affiliated Hospital of Nanjing Medical University, Nanjing 210029, China.

3State Key Laboratory of Pharmaceutical Biotechnology, Model Animal Research Center, Nanjing University, Nanjing 210061, China

4 Department of Entomology and Nematology and UC Davis Comprehensive Cancer Center, University of California, Davis, CA, 95616, USA

5Division of Clinical Neuroscience, Chiba University Center for Forensic Mental Health, 260-8670 Chiba, Japan

*Corresponding authors

Dr. Chun Yang: email address: chunyang@njmu.edu.cn

Dr. Kenji Hashimoto, email address: hashimoto@faculty.chiba-u.jp

Dr. Bruce D. Hammock, email address: [bdhammock@ucdavis.edu](http://mailto:bdhammock@ucdavis.edu/)

#These authors contributed equally to the work.

Figure S1 (Supplementary material for figure 4B)


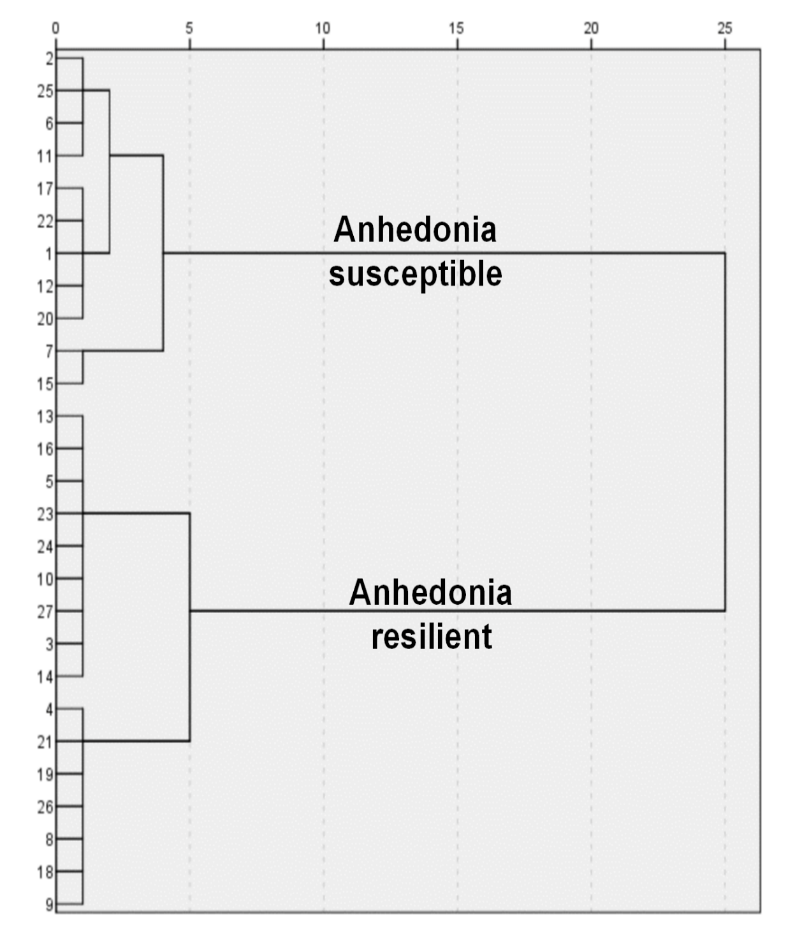


Figure S1. Dendrogram of the hierarchical clustering analysis. A total of 27 SNI mice were classified into anhedonia-susceptible and anhedonia-resilient groups by a hierarchical cluster analysis of the SPT results. SNI, spared nerve injury; SPT, sucrose preference test.

Figure S2 (Supplementary material for figure 5B)


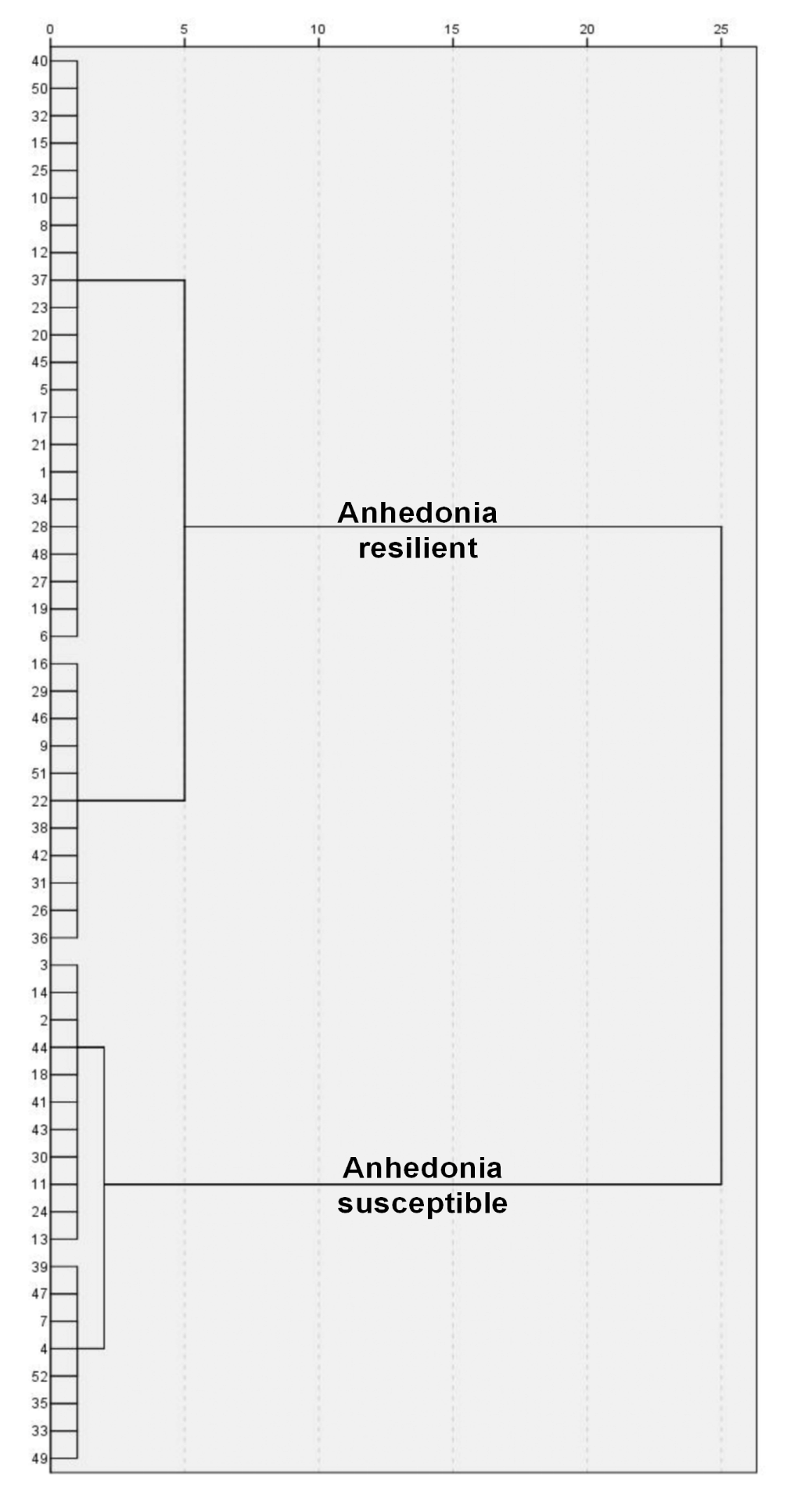


**Figure S2.** Dendrogram of the hierarchical clustering analysis. A total of 52 SNI mice were classified into anhedonia-susceptible and anhedonia-resilient groups by a hierarchical cluster analysis of the SPT results. SNI, spared nerve injury; SPT, sucrose preference test.

Table S1 (Supplementary material for figure 1B)

| Number | 1 | 2 | 3 | 4 | 5 | 6 | 7 | 8 | 9 |
| --- | --- | --- | --- | --- | --- | --- | --- | --- | --- |
| SPT | 0.70 | 0.89 | 0.80 | 0.88 | 0.63 | 0.51 | 0.78 | 0.76 | 0.61 |
| Number | 10 | 11 | 12 | 13 | 14 | 15 | 16 | 17 | 18 |
| SPT | 0.55 | 0.82 | 0.94 | 0.94 | 0.61 | 0.64 | 0.81 | 0.45 | 0.77 |

Abbreviations: SPT, sucrose preference test. Note: grey indicates anhedonia susceptible.

Table S2 (Supplementary material for figure 3B)

| Number | 1 | 2 | 3 | 4 | 5 | 6 | 7 | 8 | 9 |
| --- | --- | --- | --- | --- | --- | --- | --- | --- | --- |
| SPT | 0.57 | 1.00 | 0.65 | 0.88 | 0.87 | 0.63 | 0.60 | 0.87 | 0.66 |
| Number | 10 | 11 | 12 | 13 | 14 | 15 | 16 | 17 | 18 |
| SPT | 0.40 | 0.68 | 0.91 | 0.62 | 0.82 | 0.96 | 0.82 | 0.67 | 0.66 |
| Number | 19 | 20 | 21 | 22 | 23 | 24 | 25 | 26 | 27 |
| SPT | 0.76 | 0.92 | 0.90 | 0.93 | 0.89 | 0.84 | 0.69 | 0.85 | 0.88 |
| Number | 28 | 29 | 30 | 31 | 32 | 33 | 34 | 35 | 36 |
| SPT | 0.90 | 0.64 | 0.65 | 0.87 | 0.82 | 0.79 | 0.87 | 0.79 | 0.86 |
| Number | 37 | 38 | 39 | 40 |  | | | | |
| SPT | 0.70 | 0.61 | 0.56 | 0.85 |

Abbreviations: SPT, sucrose preference test. Note: grey indicates anhedonia susceptible.

Table S3 (Supplementary material for figure 4B)

| Number | 1 | 2 | 3 | 4 | 5 | 6 | 7 | 8 | 9 |
| --- | --- | --- | --- | --- | --- | --- | --- | --- | --- |
| SPT | 0.81 | 0.95 | 0.82 | 0.89 | 0.64 | 0.66 | 0.93 | 0.85 | 0.50 |
| Number | 10 | 11 | 12 | 13 | 14 | 15 | 16 | 17 | 18 |
| SPT | 0.87 | 0.85 | 0.73 | 0.55 | 0.83 | 0.84 | 0.82 | 0.69 | 0.49 |
| Number | 19 | 20 | 21 | 22 | 23 | 24 | 25 | 26 | 27 |
| SPT | 0.65 | 0.92 | 0.72 | 0.47 | 0.42 | 0.70 | 0.92 | 0.84 | 0.85 |
| Number | 28 | 29 | 30 | 31 | 32 | 33 |  | | |
| SPT | 0.61 | 0.93 | 0.84 | 0.90 | 0.65 | 0.68 |

Abbreviations: SPT, sucrose preference test. Note: grey indicates anhedonia susceptible.

Table S4 (Supplementary material for figure S1)

| Number | 1 | 2 | 3 | 4 | 5 | 6 | 7 | 8 | 9 |
| --- | --- | --- | --- | --- | --- | --- | --- | --- | --- |
| SPT | 0.64 | 0.61 | 0.74 | 0.93 | 0.78 | 0.57 | 0.49 | 0.84 | 0.82 |
| Number | 10 | 11 | 12 | 13 | 14 | 15 | 16 | 17 | 18 |
| SPT | 0.73 | 0.55 | 0.70 | 0.79 | 0.75 | 0.44 | 0.79 | 0.66 | 0.85 |
| Number | 19 | 20 | 21 | 22 | 23 | 24 | 25 | 26 | 27 |
| SPT | 0.87 | 0.68 | 0.96 | 0.65 | 0.78 | 0.77 | 0.61 | 0.89 | 0.72 |

Abbreviations: SPT, sucrose preference test. Note: grey indicates anhedonia susceptible.

Table S5 (Supplementary material for figure 5B)

| Number | 1 | 2 | 3 | 4 | 5 | 6 | 7 | 8 | 9 |
| --- | --- | --- | --- | --- | --- | --- | --- | --- | --- |
| SPT | 0.76 | 0.50 | 0.75 | 0.45 | 0.92 | 0.71 | 0.86 | 0.59 | 0.80 |
| Number | 10 | 11 | 12 | 13 | 14 | 15 | 16 | 17 | 18 |
| SPT | 0.52 | 0.80 | 0.54 | 0.86 | 0.61 | 0.57 | 0.85 | 0.71 | 0.42 |
| Number | 19 | 20 | 21 | 22 | 23 | 24 | 25 | 26 | 27 |
| SPT | 0.83 | 0.87 | 0.47 | 0.77 | 0.76 | 0.89 | 0.56 | 0.44 | 0.63 |
| Number | 28 | 29 | 30 | 31 | 32 | 33 | 34 | 35 | 36 |
| SPT | 0.77 | 0.59 | 0.93 | 0.59 | 0.75 | 0.41 | 0.85 | 0.49 | 0.71 |
| Number | 37 | 38 | 39 | 40 | 41 | 42 | 43 | 44 | 45 |
| SPT | 0.81 | 0.58 | 0.88 | 0.75 | 0.79 | 0.84 | 0.93 | 0.49 | 0.86 |
| Number | 46 | 47 | 48 | 49 | 50 |  |  |  |  |
| SPT | 0.66 | 0.76 | 0.87 | 0.55 | 0.47 |  |  |  |  |

Abbreviations: SPT, sucrose preference test. Note: grey indicates anhedonia susceptible.

Table S6 (Supplementary material for S2)

| Number | 1 | 2 | 3 | 4 | 5 | 6 | 7 | 8 | 9 |
| --- | --- | --- | --- | --- | --- | --- | --- | --- | --- |
| SPT | 0.88 | 0.57 | 0.64 | 0.48 | 0.93 | 0.89 | 0.41 | 0.82 | 0.68 |
| Number | 10 | 11 | 12 | 13 | 14 | 15 | 16 | 17 | 18 |
| SPT | 0.84 | 0.59 | 0.91 | 0.60 | 0.63 | 0.84 | 0.71 | 0.87 | 0.56 |
| Number | 19 | 20 | 21 | 22 | 23 | 24 | 25 | 26 | 27 |
| SPT | 0.89 | 0.82 | 0.86 | 0.67 | 0.90 | 0.58 | 0.84 | 0.76 | 0.89 |
| Number | 28 | 29 | 30 | 31 | 32 | 33 | 34 | 35 | 36 |
| SPT | 0.89 | 0.71 | 0.59 | 0.79 | 0.85 | 0.51 | 0.88 | 0.47 | 0.74 |
| Number | 37 | 38 | 39 | 40 | 41 | 42 | 43 | 44 | 45 |
| SPT | 0.91 | 0.78 | 0.43 | 0.85 | 0.59 | 0.77 | 0.59 | 0.57 | 0.92 |
| Number | 46 | 47 | 48 | 49 | 50 | 51 | 52 |  |  |
| SPT | 0.70 | 0.44 | 0.89 | 0.54 | 0.85 | 0.69 | 0.49 |  |  |

Abbreviations: SPT, sucrose preference test. Note: grey indicates anhedonia susceptible.
